# At the Edges of Photosynthetic Metabolic Plasticity—On the Rapidity and Extent of Changes Accompanying Salinity Stress-Induced CAM Photosynthesis Withdrawal

**DOI:** 10.3390/ijms22168426

**Published:** 2021-08-05

**Authors:** Michał Nosek, Katarzyna Gawrońska, Piotr Rozpądek, Marzena Sujkowska-Rybkowska, Zbigniew Miszalski, Andrzej Kornaś

**Affiliations:** 1 Institute of Biology, Pedagogical University of Krakow, Podchorążych 2, 30-084 Kraków, Poland; katarzyna.gawronska@up.krakow.pl (K.G.); andrzej.kornas@up.krakow.pl (A.K.); 2 Małopolska Centre of Biotechnology, Jagiellonian University in Kraków, Gronostajowa 7a, 30-387 Kraków, Poland; piotr.rozpadek@uj.edu.pl; 3 Department of Botany, Institute of Biology, Warsaw University of Life Sciences (SGGW), Nowoursynowska 159, 02-776 Warsaw, Poland; marzena_sujkowska@sggw.pl; 4 W. Szafer Institute of Botany, Polish Academy of Sciences, Lubicz 46, 31-512 Kraków, Poland; z.miszalski@botany.pl

**Keywords:** C_3_–CAM intermediate, common ice plant, *Mesembryanthemum crystallinum*, osmotic stress

## Abstract

The common ice plant (*Mesembryanthemum crystallinum* L.) is a facultative crassulacean acid metabolism (CAM) plant, and its ability to recover from stress-induced CAM has been confirmed. We analysed the photosynthetic metabolism of this plant during the 72-h response period following salinity stress removal from three perspectives. In plants under salinity stress (CAM) we found a decline of the quantum efficiencies of PSII (Y(II)) and PSI (Y(I)) by 17% and 15%, respectively, and an increase in nonphotochemical quenching (NPQ) by almost 25% in comparison to untreated control. However, 48 h after salinity stress removal, the PSII and PSI efficiencies, specifically Y(II) and Y(I), elevated nonphotochemical quenching (NPQ) and donor side limitation of PSI (Y_ND_), were restored to the level observed in control (C_3_ plants). Swelling of the thylakoid membranes, as well as changes in starch grain quantity and size, have been found to be components of the salinity stress response in CAM plants. Salinity stress induced an over 3-fold increase in average starch area and over 50% decline of average seed number in comparison to untreated control. However, in plants withdrawn from salinity stress, during the first 24 h of recovery, we observed chloroplast ultrastructures closely resembling those found in intact (control) ice plants. Rapid changes in photosystem functionality and chloroplast ultrastructure were accompanied by the induction of the expression (within 24 h) of structural genes related to the PSI and PSII reaction centres, including *PSAA*, *PSAB*, *PSBA* (D_1_), *PSBD* (D_2_) and *cp43*. Our findings describe one of the most flexible photosynthetic metabolic pathways among facultative CAM plants and reveal the extent of the plasticity of the photosynthetic metabolism and related structures in the common ice plant.

## 1. Introduction

Facultative CAM (Crassulacean acid metabolism) plants are plants that can induce or upregulate CAM photosynthesis in response to water-related environmental stresses (drought, high salinity). This was previously recognised as a unique trait but has been identified in a large and constantly growing group of plant families, including *Aizoaceae*, *Bromeliaceae*, *Cactaceae*, *Didiereaceae*, *Lamiaceae*, *Montiaceae* and *Vitaceae*. For most facultative CAM plants, the reversibility of stress-induced CAM photosynthesis has been confirmed [1].

*Mesembryanthemum crystallinum* L. (the common ice plant), in the *Aizoaceae* family, perfectly reflects the distinctive features of facultative CAM plants and for years was identified as a plant model in studies of osmotic stress-induced CAM photosynthesis. Elevated salinity generates a plant response that involves a wide variety of modifications resulting primarily from Na^+^ sensing and secondarily from the build-up of a toxic concentration of ions in the aerial plant parts [2,3,4]. The presence of osmoprotective mechanisms (e.g., proline synthesis and Na^+^ and Cl^–^ accumulation in bladder cells of the aerial plant parts) and the induction of antioxidative system components allows the maintenance of the main metabolic pathways in a minimally disturbed fashion, even under high-salinity conditions. In addition to the above mentioned traits, *β*-carboxylation as a part of stress-induced CAM photosynthesis greatly enhances water use efficiency. This is often recognised as a key factor allowing normal growth and development under water-related stresses. A recent study regarding the symptoms of stress-related CAM induction in the common ice plant confirmed the occurrence of *β*-carboxylation after 6 days of salt treatment [5]. The reorganisation of the CO_2_ metabolism is accompanied by substantial modifications at the chloroplast ultrastructure, photosystem reactive centre organisation and photochemical efficiency levels [6]. It was shown that in the common ice plant, salinity stress is also responsible for the enhancement of linear electron transport and the extension of sinks for reducing power [7]. In addition to these modifications of photosynthetic pathways, redox homeostasis and the osmoprotective C_3_→CAM transition are associated with changes in the structural organisation of tissues and organs [8,9].

As previously mentioned, recovery from stress-induced CAM to C_3_ photosynthesis seems to be a common trait in all known facultative CAM plants, including the ice plant, however, the rate, as well as the range of withdrawal-related changes, have been suggested to differ among them. It was shown that members of the *Talinum* and *Clusia* genera can fully stop overnight carbon fixation (distinguished as a hallmark of CAM photosynthesis) at up to 4 days following drought stress withdrawal [10]. On the other hand, for members of the *Portulacaceae* and *Aizoaceae* families, the full development of light phase-related CO_2_ fixation without overnight acidification was confirmed as early as 24 h after re-watering [10]. Our earlier study involving the withdrawal of stress-induced CAM in common ice plants showed that removal of osmotic stress inhibited nocturnal malate synthesis within 24 h. This sudden retreat from *β*-carboxylation was accompanied by a rapid decline in *PEPC1* expression [11]. The recovery of C_3_ photosynthesis was also accompanied by the reversion of the activity of the main antioxidant enzymes to a level resembling that in unstressed plants within 48 h after osmotic stress removal. The rapidity of these modifications unequivocally puts the common ice plant’s photosynthetic metabolism among those with the highest possible plasticity. According to the previously mentioned studies, photosynthetic metabolic plasticity allows for the quick adjustment to the presence and absence of stressful conditions and must play an important role in acclimation. Understanding the roles of and connection between photosynthesis and acclimation processes seems to be a top priority, especially if we take into account the fact that the ranges of many stress factors, such as high salinity, are covering an increasing area. This study aimed to demonstrate not only the speed and rate of changes occurring in response to osmotic stress withdrawal in our model facultative plant but also the extent to which these changes are related, including the changes in photochemical apparatus efficiency, chloroplast ultrastructure and the regulation of photosystem I and II structural gene expression.

## 2. Results

### 2.1. Removal of Osmotic Stress Results in Fast Recovery of PSI and PSII Efficiency

Our expectations about the time needed for stress recovery-related photosystem efficiency changes were based on our earlier study showing the full reversal of functional CAM within hours and modifications of enzymatic antioxidative system components (gene expression, protein activity) within days after osmotic stress removal. As we could not precisely predict the exact timing of such modifications in the context of photosynthetic apparatus efficiency, we decided to extend the experiment to 72 h. We performed our analyses at three consecutive time points, namely, 24, 48 and 72 h after stressor removal.

**Figure 1:**
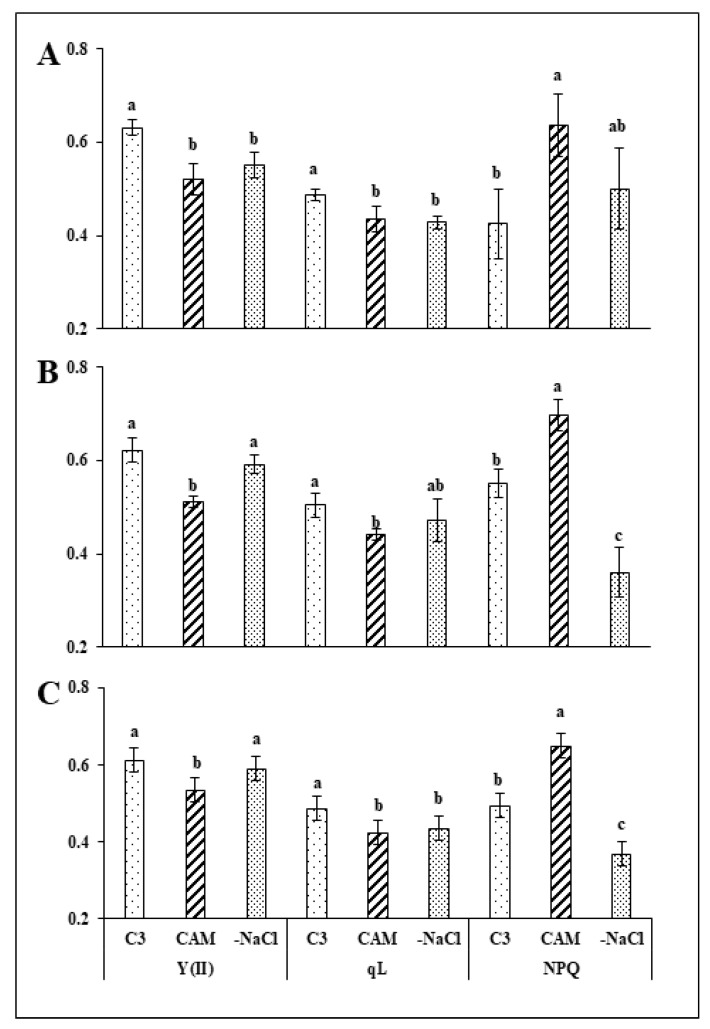
Quantum efficiency of PSII—Y(II), photochemical quenching coefficient—qL and nonphotochemical quenching—NPQ in the leaves of unstressed control (C_3_), NaCl-treated (CAM) and salt-stress withdrawn (-NaCl) *Mesembryanthemum crystallinum* L. plants measured in the middle of the light phase 24 (**A**), 48 (**B**) and 72 (**C**) hours after osmotic stress removal. Bars represent mean values (±SD) for n = 5. Different letters indicate statistically significant differences according to Tukey’s HSD test at *p* ≤ 0.05.

Earlier studies showed a clear effect of osmotic stress on photosynthetic apparatus efficiency in ice plants, including modifications of the maximal quantum yield of PSII (F_v_/F_m_), the PSII and PSI electron transport chains (ETRII, ETRI), quanta yield (Y(II), Y(I)), nonphotochemical quenching (NPQ) and donor side limitation (Y_ND_). F_v_/F_m_ was shown to be affected differently in ice plants under salinity stress (from photoinhibition to no changes at all). Therefore, we decided to concentrate our efforts on the remaining parameters, including the analysis of photochemical quenching according to the lake model (qL), as this model shows the share of photochemical processes in the energy sink. We found a decline by 18% and 12% in both the Y(II) and qL, respectively, of ice plants exposed to salt stress (CAM) in comparison to control (C_3_) at 24 h after stress removal. These changes were accompanied in salt-stressed (CAM) plants by a 50% increase in NPQ in comparison to control (Figure 1A) and were sustained in salt-stressed plants 48 and 72 h after stressor removal (Figure 1B,C). We found no substantial changes

**Figure 2:**
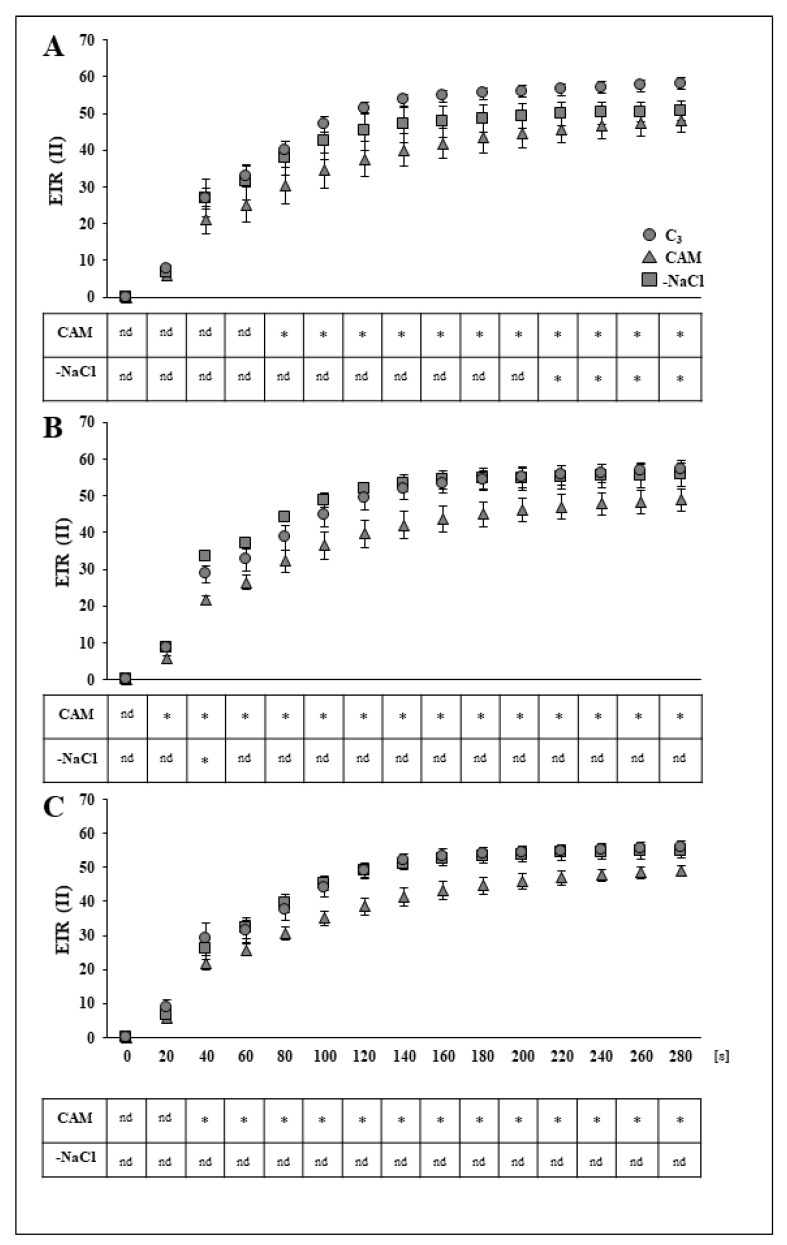
Induction curves of the PSII electron transport rate in the leaves of unstressed control (C_3_), NaCl-treated (CAM) and salt-stress-withdrawn (-NaCl) *Mesembryanthemum crystallinum* L. plants measured in the middle of the light phase 24 (**A**), 48 (**B**) and 72 (**C**) h after osmotic stress removal. Asterisks indicate a statistically significant difference in comparison to the unstressed control (C_3_) plants according to Dunnett’s test for *n* = 5; nd, no differences; [s], seconds.

In the analysed PSII parameters of stress-withdrawn plants following 24 h after stressor removal. Forty-eight hours after osmotic stress removal, the PSII quantum efficiency Y(II) reached the level measured in control plants, while the NPQ dropped below the value found in the control by almost 35%. Salt stress removal did not affect the qL of PSII. The changes observed in the Y(II) and NPQ of stress-withdrawn plants were sustained up to 72 h after stressor removal (Figure 1C). At all analysed time points in the experiment, the electron transport in photosystem II (ETRII) of plants affected with salinity stress (CAM) was inhibited in comparison to that in the control (Figure 2A–C). Removal of the stressor (-NaCl) resulted in more rapid induction of ETRII; however, the rate of induction measured in the unstressed control was achieved only 48 h after removal of the osmotic stress and was sustained further (Figure 2B,C).

**Figure 3:**
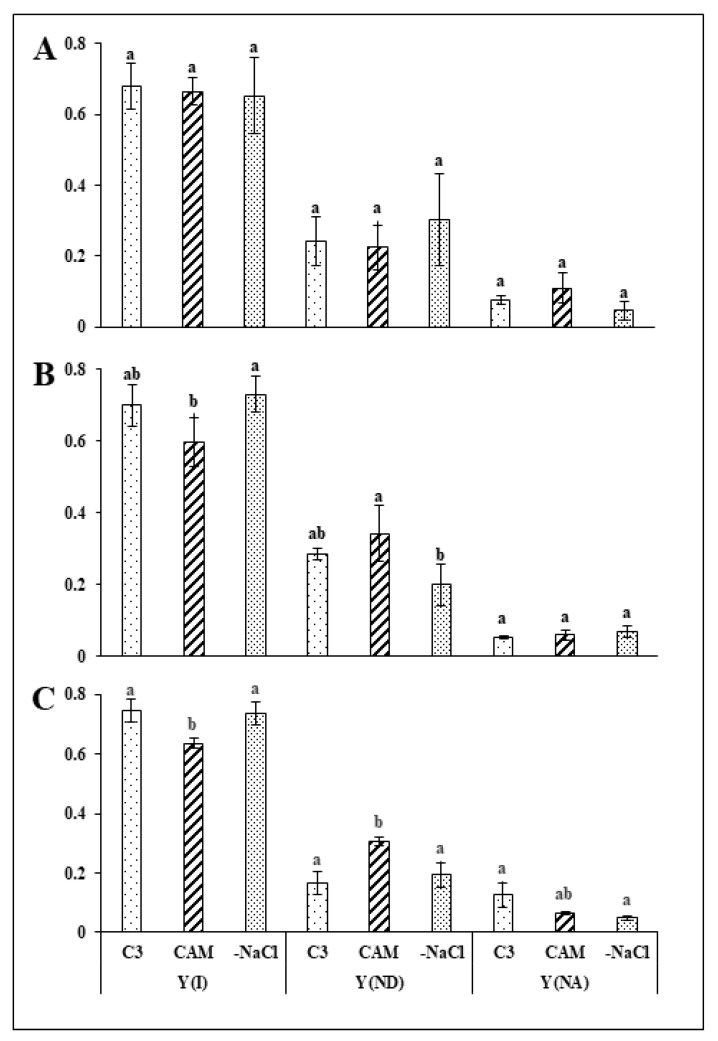
Quantum efficiency of PSI—Y(I), donor side limitation of PSI—Y_ND_ and acceptor side limitation—Y_NA_ in the leaves of unstressed control (C_3_), NaCl-treated (CAM) and salt-stress withdrawn (-NaCl) *Mesembryanthemum crystallinum* L. plants measured in the middle of the light phase 24 (**A**), 48 (**B**) and 72 (**C**) h after osmotic stress removal. Bars represent mean values (±SD) for *n* = 5. Different letters indicate statistically significant differences according to Tukey’s HSD test at *p* ≤ 0.05.

**Figure 4:**
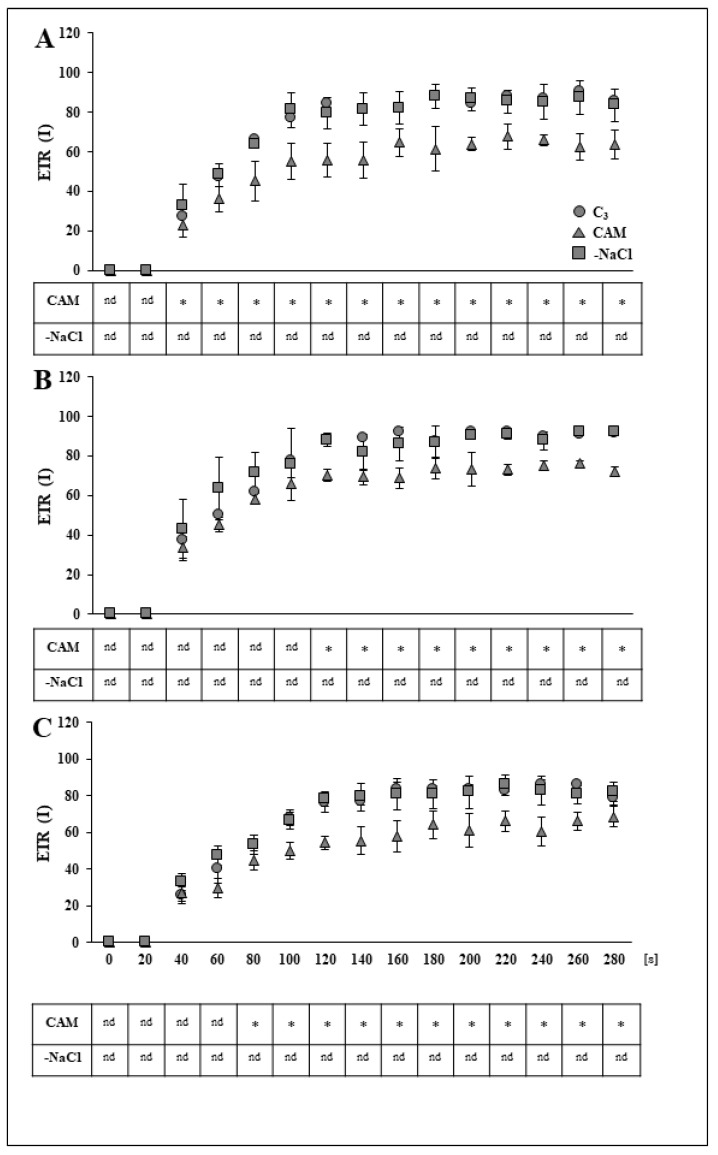
Induction curves of the PSI electron transport rate in the leaves of unstressed control (C_3_), NaCl-treated (CAM) and salt-stress withdrawn (-NaCl) *Mesembryanthemum crystallinum* L. plants measured in the middle of the light phase 24 (**A**), 48 (**B**) and 72 (**C**) h after osmotic stress removal. Asterisks indicate a statistically significant difference in comparison to unstressed control (C_3_) plants according to Dunnett’s test for *n* = 5; nd, no differences; [s], seconds.

To assess whether the effects of osmotic stress removal extended to PSI efficiency, we employed a dual PAM measurement of the quantum yield Y(I) and electron transport rate of PSI (ETRI). Unlike in the PSII analysis, we found no evidence of a detrimental effect of salt stress on the analysed parameters of PSI at up to 48 h after stressor removal (Figure 3A). However, at the last time point (72 h), we found a lower by 15% PSI quantum yield and increased by 84% PSI donor side limitation (Y_ND_) in salt-stressed (CAM) plants in comparison to unstressed plants. Salt stress had no visible effect on acceptor side limitation (Y_NA_) at any of the experimental time points. As in the PSII analysis, no substantial changes were observed in the analysed parameters (Y(II), Y_ND_) in stress-withdrawn plants 24 h after stressor removal. However, during the 48-h time frame, plants withdrawn from salt stress (-NaCl) achieved the PSI quantum yield level of the control (Figure 3B). This was accompanied by a substantial, precisely 42% decrease in PSI donor side limitation (Y_ND_) of the desalinated plants in comparison to salt-stressed plants. We found that, as in the case of PSII, the described changes were sustained for 72 h (Figure 3C). On the other hand, salt stress removal (-NaCl) did not modify the PSI acceptor side limitation Y_NA_ compared with that in the unstressed control. Similar to its effect on ETRII, salt stress substantially retarded the induction of ETRI (Figure 4A) when compared with the control plants, and this effect was sustained at subsequent experimental time points. However, contrary to ETRII PSI, electron transport in stress-withdrawn plants achieved the rate observed in the control as early as 24 h after removal of osmotic stress (Figure 4A); this effect was also sustained at 48 and 72 h after osmotic stress removal (Figure 4B,C). The trends described for the analysed PSI and PSII parameters were confirmed in most cases during the second replication of the experiment (supplementary Figures S1–S3).

### 2.2. Rapid Recovery of PSII and PSI Efficiency Is Combined with Induced Expression of Structural Genes for the Reaction Centres of Both Photosystems

**Figure 5:**
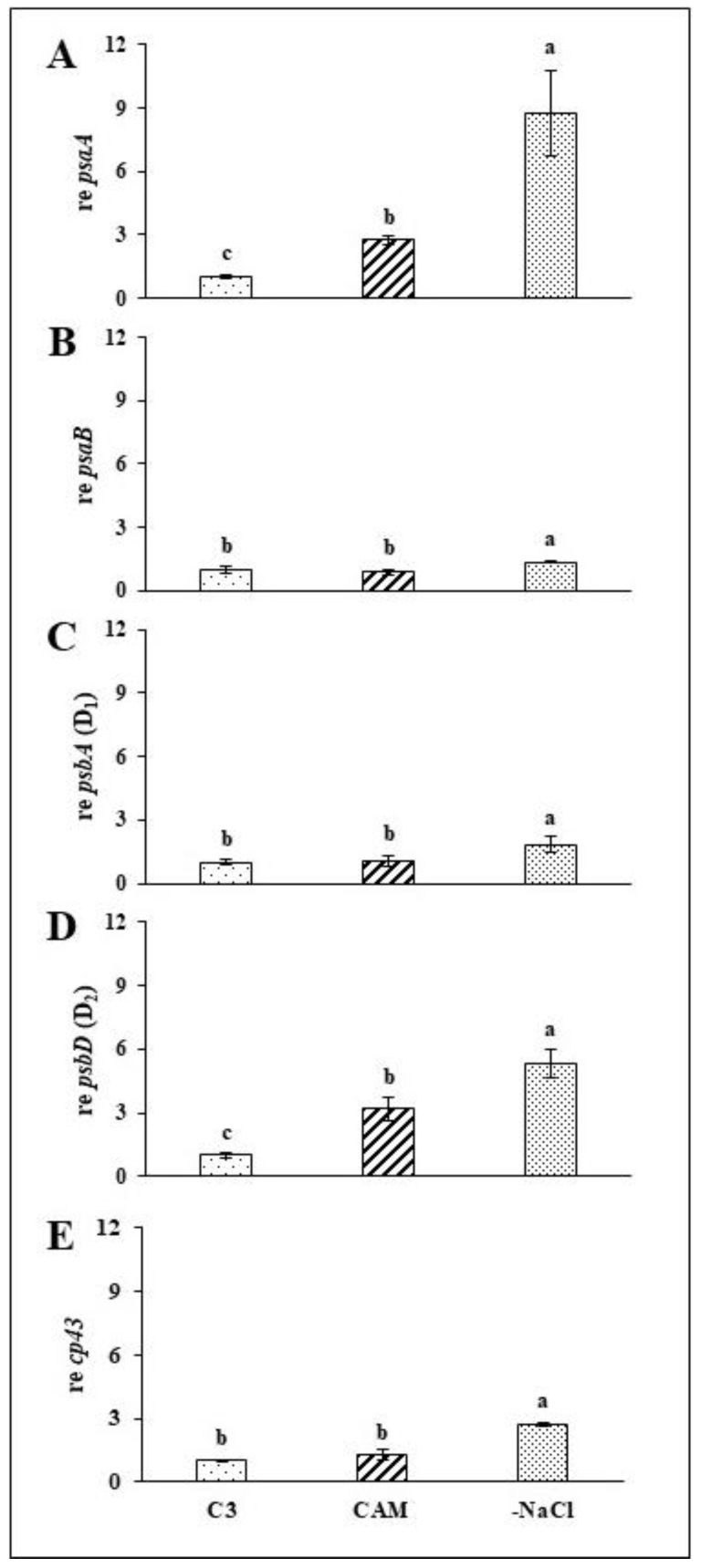
Relative expression of the PSI-A core protein of photosystem I—*PSAA* (**A**), PSI-B core subunit of photosystem I—*PSAB* (**B**), D_1_ protein of photosystem II—*PSBA* (**C**), D_2_ protein of photosystem II—*PSBD* (**D**) and cp43 protein of photosystem II—*cp43* (**E**) in the leaves of unstressed control (C_3_), NaCl-treated (CAM) and salt-stress withdrawn (-NaCl) *Mesembryanthemum crystallinum* L. plants measured 24 h after osmotic stress removal. Bars represent mean values (±SD) for *n* = 5. Different letters indicate statistically significant differences according to Tukey’s HSD test at *p* ≤ 0.05.

To assess how salt stress removal affects the expression of the structural genes associated with the reaction centres of PSI and PSII, we analysed the abundance of their respective transcripts with *q*PCR during a 72-h recovery period following stress removal. In all studied genes, substantial modifications related to osmotic stress withdrawal were observed during the first 24 h of recovery. We found over 2-fold and over 3-fold increases in *PSAA* (PSI-A core protein of PS I) and *PSBD* (D_2_ protein of PS II) expression in salt-stressed (CAM) plants in comparison to intact plants (Figure 5A,D). Except for *PSAA* and *PSBD*, the expression of the analysed structural genes of the photosystem centres remained mostly unaffected during salt stress (Figure 5B,C,E). On the other hand, we found that *PSAA* was the gene mostly affected by osmotic stressor withdrawal, with expression over 3-fold and over 8-fold higher than that in intact (C_3_) and stressed (CAM) plants, respectively (Figure 5A). The expression of *CP43* was upregulated in a similar manner, with an over 2-fold increase compared with that in both intact (C_3_) and salt-stressed (CAM) plants (Figure 5E). The expression of *PSAB* (PSI-B core subunit of PS I) and *PSBA* (D_1_ protein of PS II) was modified in the same direction; however, in both cases, the upregulation related to osmotic stress removal was less intense than that of *PSAA*, *PSBD* and *cp43* (Figure 5B,C). The upregulated expression of *PSBD* due to salt stress was enhanced even further with osmotic stress removal, resulting in an over 1,5-fold increase in comparison to that in salt-stressed (CAM) plants (Figure 5D). All modifications described here at the 24-h experimental time point were sustained at 48 and 72 h after stressor removal (supplementary Figure S4).

### 2.3. Withdrawal from Osmotic Stress Is Accompanied by the Rapid Reorganisation of Chloroplast Ultrastructure

**Figure 6:**
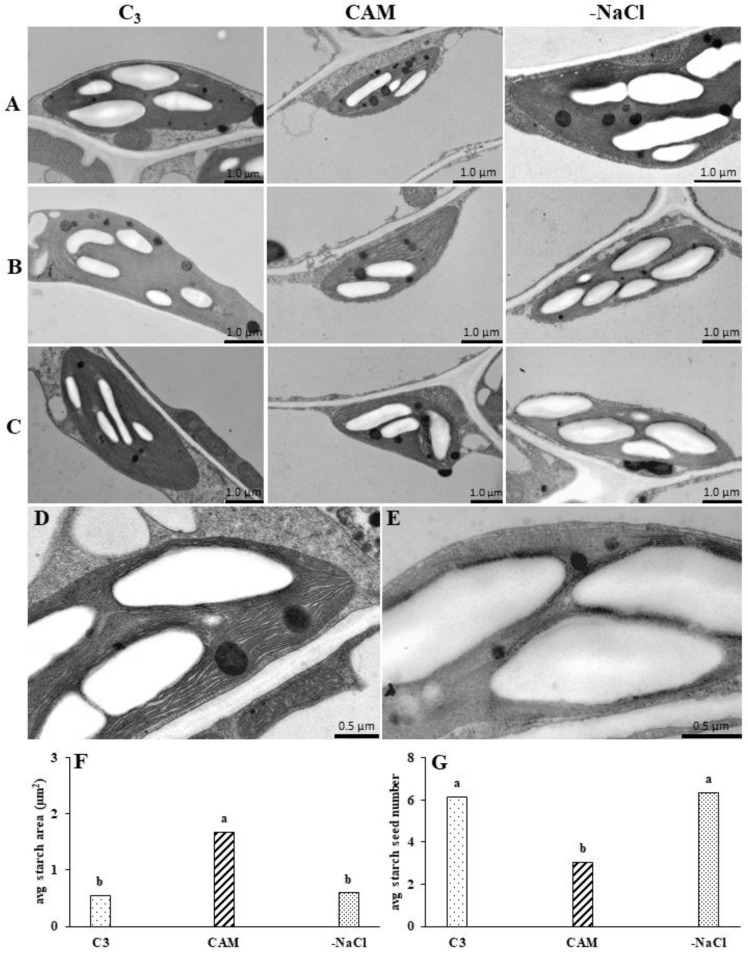
Electron micrographs of chloroplasts in unstressed control (C_3_), NaCl-treated (CAM) and salt-stress withdrawn (-NaCl) *Mesembryanthemum crystallinum* L. plants taken in the middle of the light phase 24 (**A**), 48 (**B**) and 72 (**C**) h after osmotic stress removal. To clearly display the ultrastructural changes, sections (**D**,**E**) of the panel show the thylakoid membranes of NaCl-treated (CAM) and salt-stress withdrawn (-NaCl) plants, respectively, taken at higher magnification. The presented micrographs are representative examples of at least 23 repetitions. Sections (**F**,**G**) show the average area (μm^2^) and the number of starch grains, respectively, assessed through an image analysis of chloroplast micrographs of unstressed control (C_3_), NaCl-treated (CAM) and salt-stress withdrawn (-NaCl) *Mesembryanthemum crystallinum* L. plants 24 h after osmotic stress removal. Bars represent mean values (±SD) for *n* = 23. Different letters indicate statistically significant differences according to Tukey’s HSD test at *p* ≤ 0.05.

To determine the range of modifications resulting from osmotic stress withdrawal, we analysed the chloroplast ultrastructure of salt-stressed and salt stress-withdrawn plants. In the chloroplasts of intact (C_3_) plants, we found densely packed, intact thylakoids that were unstacked or organised in irregular grana stacks located between small- and medium-sized starch grains (Figure 6A–C). In response to osmotic stress, the thylakoid membrane system was reorganised. In the chloroplasts of salt-stressed plants, we found swollen thylakoids that were mostly unstacked and localised between large starch grains (Figure 6D).

We found that osmotic stress increased the size and reduced the average starch grain quantity over 3-fold and 2-fold, respectively (Figure 6F,G). In plants withdrawn from osmotic stress, thylakoid swelling was barely visible. This effect was accompanied by the reorganisation of the membrane system, which began to resemble the one observed in the untreated control, with densely packed thylakoids organised mostly in irregular grana (Figure 6E). The changes resulting from osmotic stress removal were also expressed in the reduced area and increased number of starch grains, similar to those found in unstressed (C_3_) plants. The ultrastructural modifications observed 24 h after osmotic stress removal were sustained at 48 and 72 h (Supplementary Figure S5).

## 3. Discussion

### 3.1. Rapid Modifications in PSII and PSI Functionality during Recovery from Osmotic Stress Confirm the Great Flexibility of the Common Ice Plant Photosynthetic Apparatus

An earlier study on the functionality of the photosynthetic apparatus in *M.* *crystallinum* showed higher and similar quantum efficiencies of PSII and PSI, respectively, in salt-stressed (CAM) plants than in control (C_3_) plants [5]. Our analysis revealed quite a different picture of PSII and PSI functionality during a similar salt treatment, with lower PSII and PSI quantum efficiencies, elevated nonphotochemical quenching (NPQ), decreased photochemical quenching (qL) and elevated acceptor side limitation (Y_ND_) of PSI detected at midday in salt-stressed plants. We also found retarded photosynthetic electron transport (PET) in both photosystems in salt-stressed plants. Most of these modifications of PSII and PSI functionality were previously found to be a part of salt stress-induced CAM photosynthesis [12,13]. Moreover, it was suggested that despite the high salt tolerance of *M. crystallinum*, the plants still experience salt stress, and the resultant CAM induction was reported to be a source of photoinhibition [12,13,14,15]. Although we could not confirm the occurrence of photoinhibition in our study, we suggest that salt-induced CAM plants struggled with excess energy that could not be quenched with photochemical processes. We believe that the substantially lower amount of RubisCO (ribulose-1,5-bisphosphate carboxylase-oxygenase) previously detected in salt-stressed plants than in C_3_ plants [6,9] may to some extent explain the retardation of the photochemical processes of PSII and PSI. Photochemical quenching is at its highest at midday due to elevated RubisCO activity resulting from the abundance of CO_2_. An osmotic stress-induced decline in the amount of RubisCO results in a substantially lower capacity for electron sinkage as well as a lower energy demand, which may be responsible for the declines observed in the functionalities of both PSII and PSI and the necessity of excess energy dissipation through nonphotochemical mechanisms. It was recently confirmed that the largest share of NPQ build-up accompanying salinity-induced CAM photosynthesis belonged to heat dissipation through antennae [13]. Among CAM facultative species, those with increasingly flexible photosynthetic metabolisms can be distinguished. It was shown that the return from nocturnal (CAM photosynthesis) to diel (C_3_ photosynthesis) CO_2_ fixation may be re-established in different facultative CAM plants as a response to stress withdrawal within as early as 24 h (*Portulaca oleracea*, *M. crystallinum* L.) and even up to 96 h (*Talinum triangulare*, *Clusia pratensis*) [10]. Our previous study involving salinity stress withdrawal in *M. crystallinum* confirmed these findings. We discovered substantial downregulation of *pepc1* (an osmotic stress-related member of the PEPC gene family) expression during the first hours after osmotic stress removal. This effect was accompanied by the re-established Δ malate, a hallmark of functional CAM photosynthesis that, in salt stress-withdrawn plants, rapidly (24 h) returned to the values typical for C_3_ plants [11]. Here, we showed that the PSII and PSI functionality of intact (C_3_) plants was quickly achieved by salt-stressed withdrawn plants. Most of the disturbances found in PSII and PSI functionality disappeared within 48 h after osmotic stress removal. It can be hypothesised that one of the reasons for the rapid return of the functionality of both photosystems was the restoration of the content and activity of RubisCO characteristic of C_3_ plants. Altogether, our findings show that rapid modifications of carbon metabolism-related genetic (*PEPC1* expression) and biochemical (CO_2_ fixation, malate concentration build-up, tissue acidification) pathways resulting from the withdrawal of osmotic stress are coupled with modifications of photosynthetic apparatus performance.

### 3.2. Expression of PSII and PSI Structural Genes Is Rapidly Modified in Response to Osmotic Stress Absence

The regulation of photosynthesis-related genes during salinity stress remains a poorly understood phenomenon in both salt-sensitive (glycophyte) and salt-tolerant (halophyte) plants. It has been shown that in glycophytes, salt stress is responsible mostly for photosynthesis-related gene downregulation [16]. Large-scale analysis of the common ice plant transcriptome under salt stress revealed an increase in the abundance of genes related to CAM photosynthesis [17]. Our analysis showed that in addition to inducing transcripts of *PSAA* (PSI-A core protein of PS I) and *PSAD* (D_2_ protein of PS II), the regulation of the main structural genes of PSII and PSI remained unaffected by salt stress. To some extent, this result correlates with an earlier study [6], and the regulation of photosystem gene expression during the salt stress response seems to rely on a combination of different signals. Two of these signals may have opposite effects; to meet the high demand for de novo synthesis of photosystem proteins resulting from stress-related protein degradation, the upregulation of their respective genes is required. On the other hand, it was previously proposed that a decline in PET is responsible for photosystem gene downregulation [18]. Our results show that salt stress had a rather minimal impact, however, the withdrawal of salt stress strongly induced the expression of all analysed genes. According to the previously mentioned studies, it can be suggested that the rapid induction of these genes is stimulated not only by the recovered efficiency of both photosystems and PET but also to supply the high demand for de novo synthesis of photosystem proteins.

### 3.3. Rapid Changes in PSII and PSI Functionality Are Accompanied by Chloroplast Ultrastructure Modification during Osmotic Stress Recovery

Ion homeostasis disorder accompanying salt stress is responsible for alterations in chloroplast ultrastructure, including thylakoid membrane swelling, grana thylakoid lamellae disorder, the fracturing of stroma thylakoid lamellae and chloroplast membrane disintegration; these effects have been confirmed in both glycophytes and halophytes of different plant species [6,19,20,21]. To present the extent of changes resulting from osmotic stress withdrawal, we analysed the chloroplast ultrastructure of plants that had recovered from salinity stress. Our analysis of the salt-stressed (CAM) chloroplast ultrastructure confirmed the presence of swollen thylakoids organised mostly in unstacked grana [22]. It is possible that the salt-induced decline in photochemical efficiency observed, especially in PSII, was related not only to the decreased amount of RubisCO but also to the detrimental effects of salt stress on the thylakoid ultrastructure. Although it was previously suggested that thylakoid swelling might not be related to salt stress occurrence [6], the strong reduction in swelling observed in our experiments during the first 24 h of recovery indicates a relationship with osmotic stress. Modifications of the thylakoid membrane system during the salt stress response in common ice plants are accompanied by starch remobilisation. It was previously shown in the common ice plant that increased daily starch turnover resulting from the induced activity of *α*- and *β*-amylases, the main starch-degrading enzymes, was unequivocally related to salt stress [23]. In CAM-performing plants, the products of transitory starch degradation supply the cytosol with the carbohydrates required for the synthesis of phospho*enol*pyruvate (PEP), a substrate in primary CO_2_ fixation, by phospho*enol*pyruvate carboxylase (PEPC). In isolated chloroplasts supplied with oxaloacetic acid (OAA), a substrate of malate synthesis by plastidic malate dehydrogenase (MDH), the rate of starch degradation was increased [24]. Our analysis confirmed the salinity-induced degradation of starch. Additionally, stress-related starch degradation was confirmed by the increased number of starch grains found in the chloroplasts recovered (24 h) from osmotic stress. Taken together, our results show that the flexibility of ice plants’ carbon metabolism also extends to the chloroplast ultrastructure.

## 4. Materials and Methods

### 4.1. Plant Material

*Mesembryanthemum crystallinum* L. seeds (provided by the Technical University, Darmstadt, Germany) were sown onto a soil substrate in a greenhouse under 300–350 µmol photons m^–2^·s^–1^ of photosynthetically active radiation (PAR), a 16/8 h day/night photoperiod, a day/night thermoperiod of 25/17 °C and 55–65% relative humidity (RH), as previously described [25]. The substrate implemented in the experiment was made of market available soil (“Athena“ Bio-Products, Golczewo, Poland; pH 6.75; d = 0.24 kg dm^−^^3^) and sand (grain size in the range of 1–2 mm) mixed in a 4:1 *v*/*v* ratio. Two weeks after sowing, each seedling with a fully developed 2nd leaf pair was transferred to an individual 0.4-L pot with 360 ± 0.1 g of mentioned substrate applied per each pot. After 6 weeks, the plants were divided into two groups: the first group was irrigated with tap water (C_3_, intact control plants), and the second group was irrigated with 0.4 M NaCl (CAM, salt-stressed plants). After 14 days, CAM development in the salt-stressed plants was confirmed by the measurement of the diurnal ∆ malate, a hallmark of functional CAM photosynthesis expressed as the difference in cell sap malate concentration between the beginning and the end of the light phase. ∆ malate was measured according to the method previously described for *Clusia hilariana* Schltdl [26]. The next day, half of the CAM-performing plants were subsequently subjected desalinisation process by continuous rinsing of the soil substrate with tap water for 2 h (-NaCl, salt stress-withdrawn plants). Fluorometric measurements, as well as the collection of C_3_, CAM and NaCl plant leaves for ultrastructure and molecular analysis, were performed at midday at 24, 48 and 72 h after osmotic stress removal. For the analysis of gene expression in leaves, frozen (LN_2_) leaf tissue was ground to a fine powder in LN_2_ and then stored at −80 °C until further analysis.

### 4.2. Quantum Efficiencies of PSII and PSI

PSII and PSI photochemistry was analysed simultaneously with a Dual PAM 100 (Heinz Walz GmbH, Effeltrich, Germany) fluorescence system. Induction curves were obtained from dark-adapted (20 min) plants, and each experimental variant was measured in 5 repetitions during two independent experiments. The minimal fluorescence yield (F_0_) was measured at less than 1 μmol photons m^–2^·s^–1^ intensity, whereas the maximum fluorescence yield (F_m_) was measured after the application of a 1 s. saturating pulse of 2500 μmol photons m^–2^·s^–1^. After dark relaxation (45 s), the centres were oxidised under red-orange actinic light with an irradiance of 126 μmol photons m^–2^·s^–1^. After 260 s of light adaptation, the current (F_t_) and maximum (F_m′_) light-adapted fluorescence were measured. The PSII parameters were calculated using the following Equations (1–4).

Y(II) = (F_m’_ − F)/F_m’_ (1)

qL = (F_m’_ − F)/(F_m’_ − F_0’_) × F_0’_/F = qP × F_0’_/F (2)

NPQ = (F_m_−F_m’_)/F_m’_ (3)

F_0’_ = F_0_/(F_v_/F_m_ + F_0_/F_m’_) (4)

Y(II) was calculated according to [27]. Calculation of photochemical quenching (qL) coefficient was delivered by PAM 100 (Heinz Walz GmbH, Effeltrich, Germany) user manual. Non-photochemical quenching (NPQ) and F_0_ calculations were performed according to [28] and [29], respectively.

The parameters describing PSI efficiency were calculated as described in the Dual-PAM 100 (Walz, Germany) user manual. The photochemical quantum yield of PSI-Y(I) was calculated from the complementary PSI quantum yields using the following Equation (5), namely, the nonphotochemical energy dissipation, Y_ND_ and Y_NA_.

Y(I) = 1 − Y_ND_ − Y_NA_ (5)

The donor side limitation of PSI-Y(ND), was calculated from the reduced P700 using the following Equation (6), according to the manufacturer’s manual.

Y_ND_ = 1 − P_700 red_ (6)

Similarly, Y_NA_, representing the acceptor side limitation of PSI, was determined as the fraction of the P700 centres that could not be oxidised with a saturation pulse calculated according to the following Equation (7).

Y_NA_ = (P_m_ − P_m__′_)/P_m_ (7)

where P_m_ and P_m__′_ represent the maximal change in the P700 signal upon the application of a saturation pulse in the dark-adapted state and light state, respectively.

### 4.3. RNA Preparation

Total RNA was isolated with an Aurum™ Total RNA Mini Kit (Bio-Rad, Hercules, CA, USA) according to the method previously described [30]. For the removal of DNA contamination, digestion with DNase I (DNA I Amplification Grade, Merck, Darmstadt, Germany) was used. RNA purity and quantity were determined using a Biospec-Nano (Shimadzu, Japan). To assess the integrity and purity of the RNA, the extracted RNA was separated by electrophoresis on agarose (1.5%) gels stained with EtBr. The bands were visualised on a Molecular Imager^®^ ChemiDoc™ XRS+ Imaging System (Bio-Rad, Hercules, CA, USA).

### 4.4. qPCR

Reverse transcription was carried out on 1000 ng of total RNA with an iScript cDNA Synthesis Kit (Bio-Rad, Hercules, CA, USA). During qPCR, the samples were labelled with iQ™ SYBR^®^ Green Supermix (Bio-Rad, Hercules, CA, USA) fluorescent dye. For a single reaction, 10–15 ng of cDNA and 150 nM of gene-specific primers were used. To test the amplification specificity, a dissociation curve was acquired by heating samples from 60 °C to 95 °C. Polyubiquitin was used as a housekeeping reference gene. The reaction efficiency was tested by serial dilutions of cDNAs with gene-specific primers (Supplementary Table S1). The expression was calculated from at least three reactions with unstressed control (C_3_) plants after 24 h as calibrators according to a previously described method [31].

### 4.5. Chloroplast Ultrastructure—TEM Analysis

For (ultra)structural studies, collected leaf fragments were fixed in a mixture of 4% paraformaldehyde and 5% glutaraldehyde in 0.1 M sodium cacodylate buffer, pH 7.3, for 2 h at room temperature and air pressure (0.3 hPa), washed with 0.1 M cacodylate buffer, and postfixed for 2 h at 4 °C in 1% osmium tetroxide in 0.1 M cacodylate buffer. Then, the samples were dehydrated in an ethanol series at room temperature, rinsed three times in propylene oxide, embedded in epoxy resin Epon 812 (Fluka, Buchs, Switzerland) and polymerised for 24 h at 60 °C. Ultrathin sections collected on formvar-coated grids were briefly stained with uranyl acetate and lead citrate and examined under an FEI 268D ‘Morgagni’ transmission electron microscope (FEI-Thermo Fisher Scientific, Waltham, MA, USA) operating at 80 kV and equipped with a ‘Morada’ digital camera (Olympus-SIS, Tokyo, Japan). The collected digital microscopic images were saved as.jpg files and, if necessary, processed in Photoshop CS 8.0 (Adobe Systems, Mountain View, CA, USA) software with non-destructive tools (contrast and/or levels).

### 4.6. Image Analysis of Electron Micrographs

Image analysis of the chloroplast micrographs was performed with ImageJ 2 (under a GPL licence; NIH, Bethesda, MD, USA).

### 4.7. Statistical Analysis

The results were analysed with Statistica 13.3 (TIBCO, Palo Alto, CA, USA) statistical software. For the determination of statistically significant differences in the electron transport rates of PSII and PSI between experimental variants and the control, Dunnett’s test was applied. One-way ANOVA followed by a post hoc Tukey HSD test was used to evaluate individual treatment effects at *p* ≤ 0.05.

## 5. Conclusions

Here presented results show that the presence of salinity stress is required not only for the induction of stress-dependent CAM photosynthesis but also for maintaining its functioning. The rapid shutdown of the energy-demanding functional CAM seems to be one small component of the flexibility features. As we showed here, the metabolic flexibility of the common ice plant includes rapid and far-reaching changes. This includes photosystem high-performance recovery, the induction of the expression of reaction centre structural genes and the reorganisation of the chloroplast ultrastructure, and most of the mentioned modifications are completed within 24 h after osmotic stress removal. Here, we confirmed that the photosynthetic metabolism of the common ice plant may be one of the most highly plastic processes documented among facultative CAM plants.

## Data Availability

Data is contained within the article or supplementary material.

## Supplementary Materials

The following are available online at https://www.mdpi.com/article/10.3390/ijms22168426/s1.

## Abbreviations

CAM	Crassulacean acid metabolism;
ETRI	Electron transport chain of PSI;
ETRII	Electron transport chain of PSII;
F_0_	Minimal fluorescence yield;
F_m_	Maximum fluorescence yield;
F_m′_	Maximum light-adapted fluorescence;
F_v_/F_m_	Maximum quantum yield of PSII;
MDH	Malate dehydrogenase;
NPQ	Non-photochemical quenching;
OAA	Oxaloacetic acid;
PAR	Photosynthetically active radiation;
PEP	Phospho*enol*pyruvate;
PEPC	Phospho*enol*pyruvate carboxylase;
PET	Photosynthetic electron transport;
*PSAA*	PSI-A core protein of PS I;
*PSBD*	D2 protein of PS II;
PSI	Photosystem I;
PSII	Photosystem II;
qP, qL	Photochemical quenching calculated based on the puddle and lake model, respectively;
RuBisCO	Ribulose-1,5-bisphosphate carboxylase-oxygenase;
Y(I)	Quantum yield of (PSI);
Y(II)	Quantum yield of (PSII);
Y_NA_	Quantum yield of energy dissipation due to acceptor side limitation in PSI;
Y_ND_	Quantum yield of energy dissipation due to donor side limitation in PSI.
