# Supplementary material for: At the Edges of Photosynthetic Metabolic Plasticity—On the Rapidity and Extent of Changes Accompanying Salinity Stress-Induced CAM Photosynthesis Withdrawal
Source: Int J Mol Sci. 2021 Aug 5;22(16):8426. doi: 10.3390/ijms22168426 (PMC8395101; doi:10.3390/ijms22168426)
Supplement: Supplementary file 1 [file ijms-22-08426-s001.zip › ijms-1291654-supplementary.pdf]

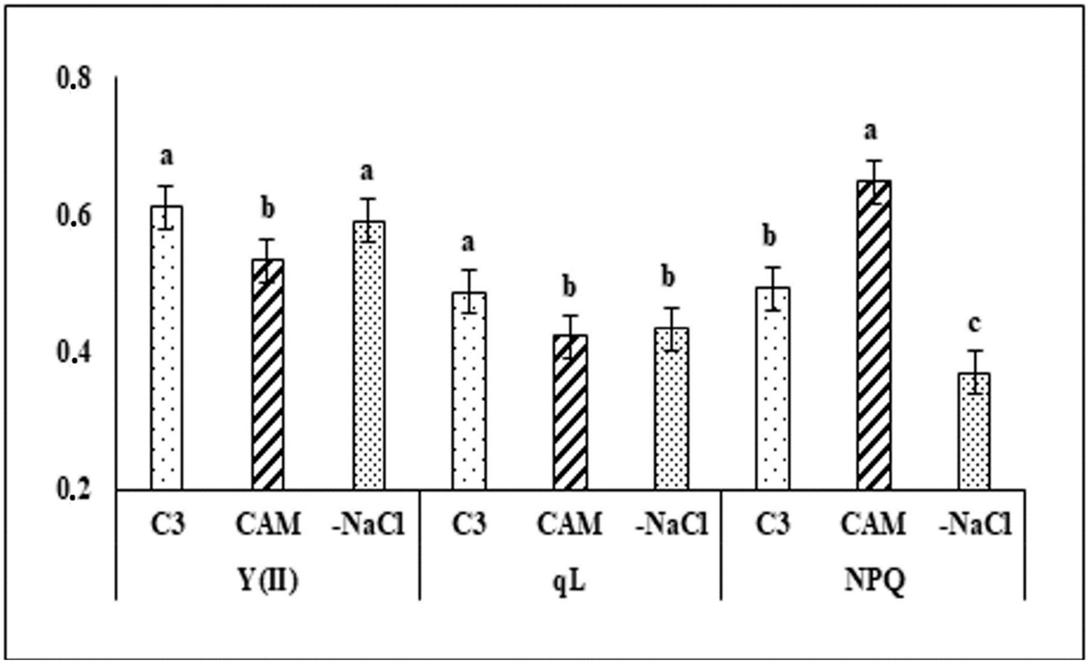

Figure S1. Second replication of the experiment. Quantum efficiency of PSII—Y(II), photochemical quenching coefficient—qL and non-photochemical quenching—NPQ in leaves of unstressed control (C_3), NaCl-treated (CAM) and salt-stress withdrawn (-NaCl) *Mesembryanthemum crystallinum* L. plants measured in the middle of the light phase 72 hours past osmotic stress removal. Bars represent mean values (±SD) for n=5. Different letters indicate statistically significant differences according to Tukey's HSD test at $p \leq 0.05$.

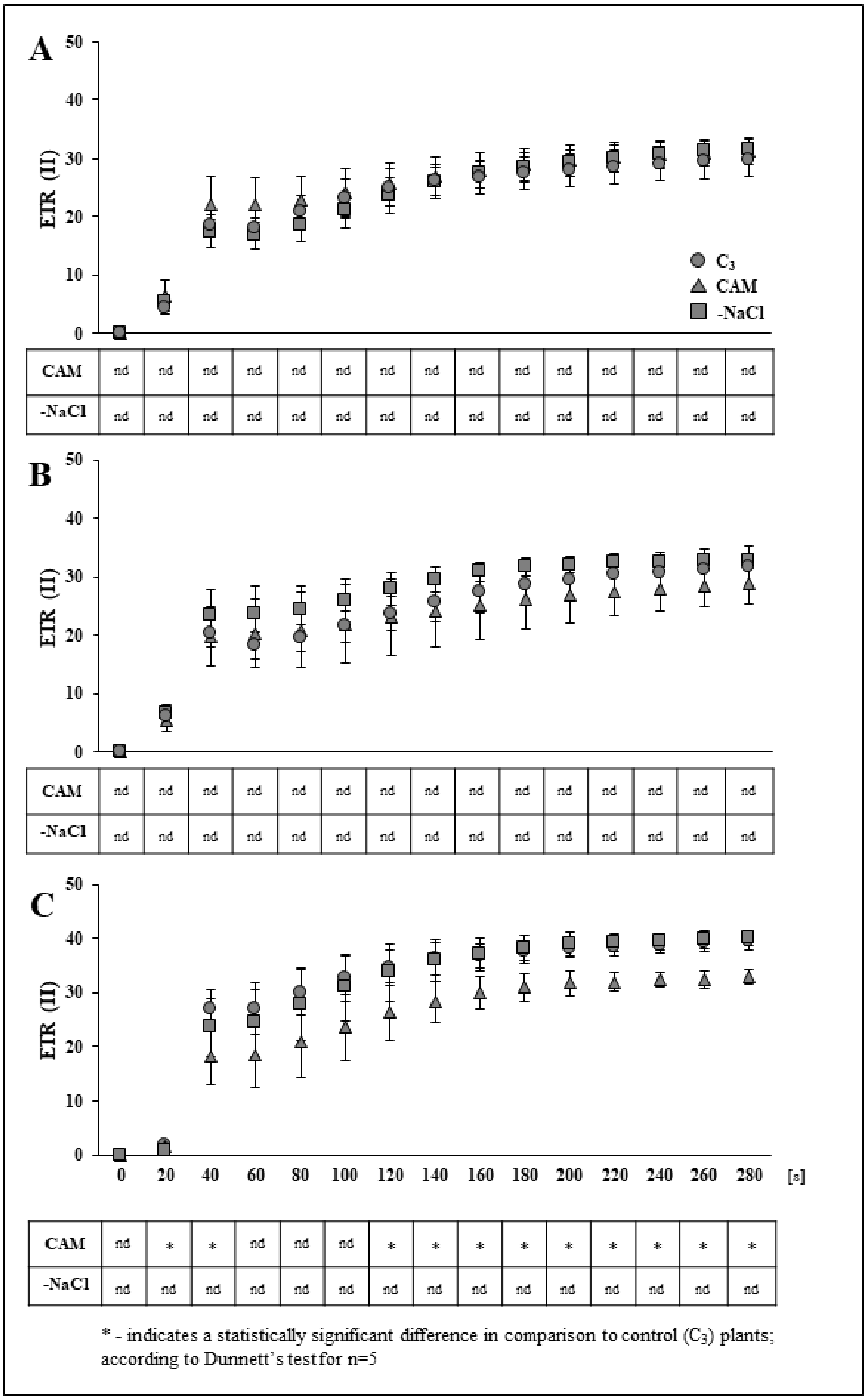

Figure S2. Second replication of the experiment. Induction curves of the PSII electron transport rate in leaves of unstressed control (C_3), NaCl-treated (CAM) and salt-stress-withdrawn (-NaCl) *Mesembryanthemum crystallinum* L. plants measured in the middle of the light phase 24 (**A**), 48 (**B**) and 72 (**C**) hours past osmotic stress removal. Asterisk indicates a statistically significant difference in comparison to unstressed control (C_3) plants according to Dunnett's test for *n*=5. nd, no differences; [s], seconds

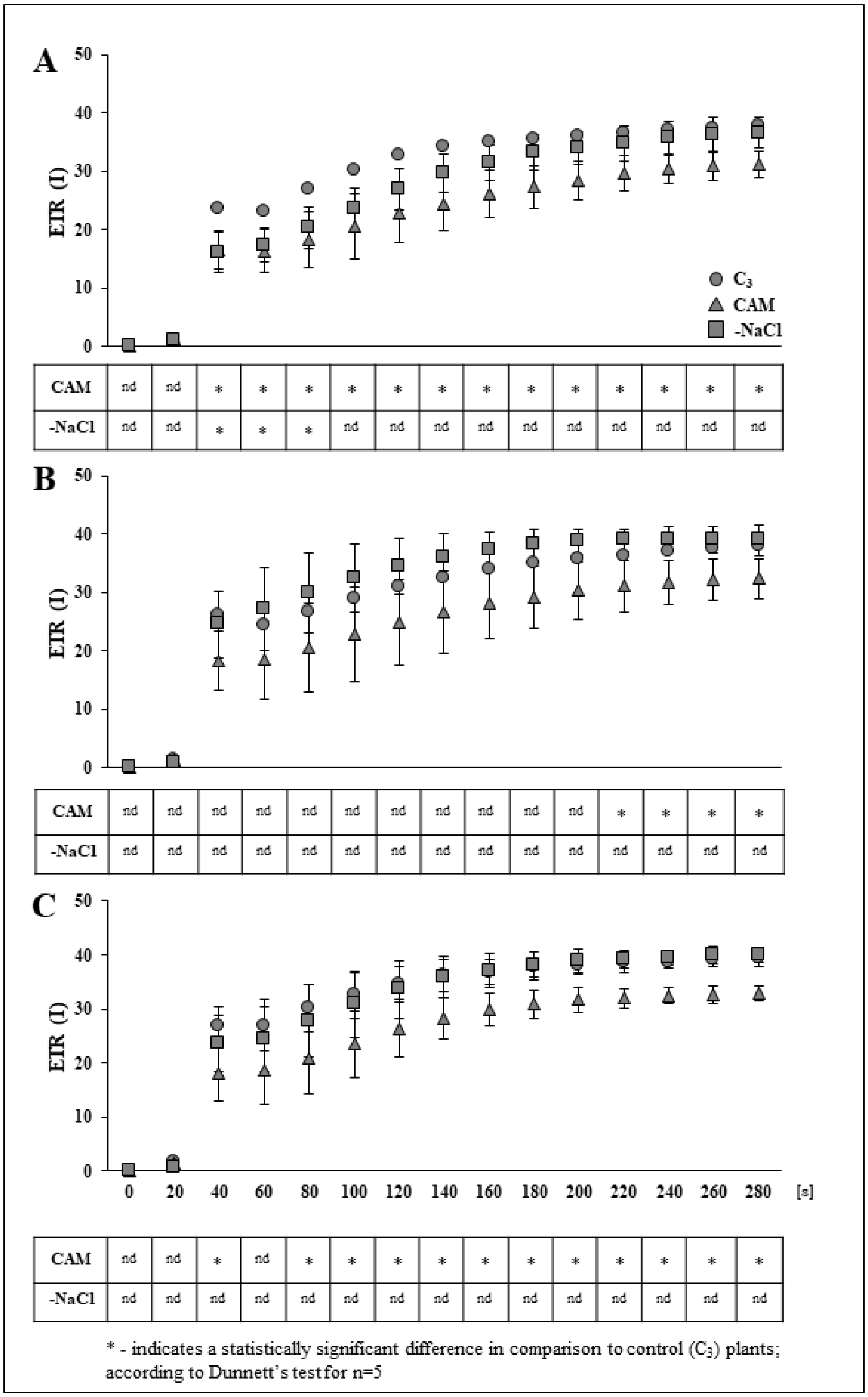

A

	0	20	40	60	80	100	120	140	160	180	200	220	240	260	280
CAM	nd	nd	*	*	*	*	*	*	*	*	*	*	*	*	*
-NaCl	nd	nd	*	*	*	nd	nd	nd	nd	nd	nd	nd	nd	nd	nd

B

	0	20	40	60	80	100	120	140	160	180	200	220	240	260	280
CAM	nd	nd	nd	nd	nd	nd	nd	nd	nd	nd	nd	*	*	*	*
-NaCl	nd	nd	nd	nd	nd	nd	nd	nd	nd	nd	nd	nd	nd	nd	nd

C

	0	20	40	60	80	100	120	140	160	180	200	220	240	260	280
CAM	nd	nd	*	nd	*	*	*	*	*	*	*	*	*	*	*
-NaCl	nd	nd	nd	nd	nd	nd	nd	nd	nd	nd	nd	nd	nd	nd	nd

Figure S3. Second replication of the experiment. Induction curves of the PSI electron transport rate in leaves of unstressed control (C_3), NaCl-treated (CAM) and salt-stress withdrawn (-NaCl) *Mesembryanthemum crystallinum* L. plants measured in the middle of Table 24. (**A**), 48 (**B**) and 72 (**C**) hours past osmotic stress removal. Asterisk indicates a statistically significant difference in comparison to unstressed control (C_3) plants according to Dunnett's test for n=5; nd, no differences; [s], seconds

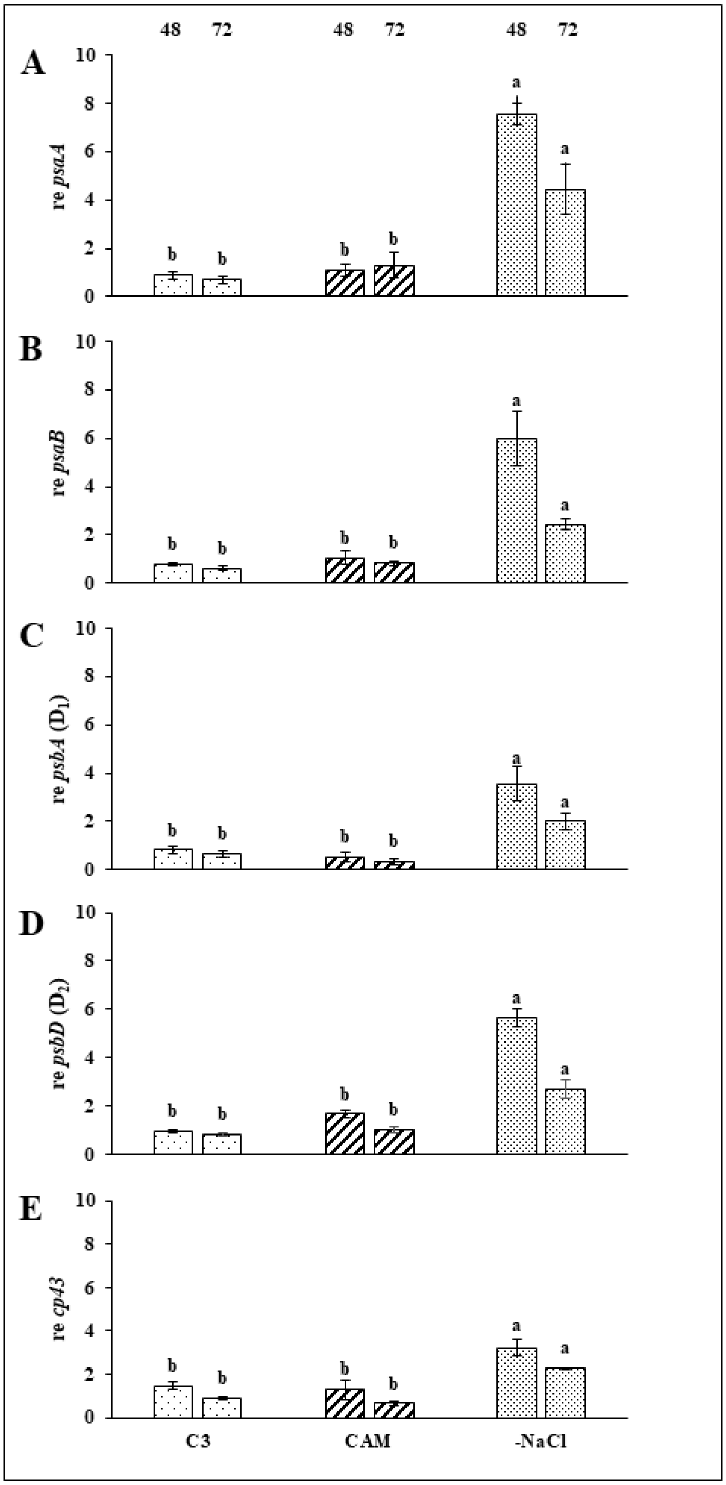

Figure S4. Second replication of the experiment. Relative expression of PSI-A core protein of photosystem I—*PSAA* (**A**), PSI-B core subunit of photosystem I—*PSAB* (**B**), D_1 protein of photosystem II—*PSBA* (**C**), D_2 protein of photosystem II—*PSBD* (**D**) and cp43 protein of photosystem II – *cp43* (**E**) in leaves of unstressed control (C_3), NaCl-treated (CAM) and salt-stress withdrawn (-NaCl) *Mesembryanthemum crystallinum* L. plants measured 48 and 72 hours past osmotic stress removal. Bars represent mean values (±SD) for *n*=5. Different letters indicate statistically significant differences according to Tukey's HSD test at $p \leq 0.05$.

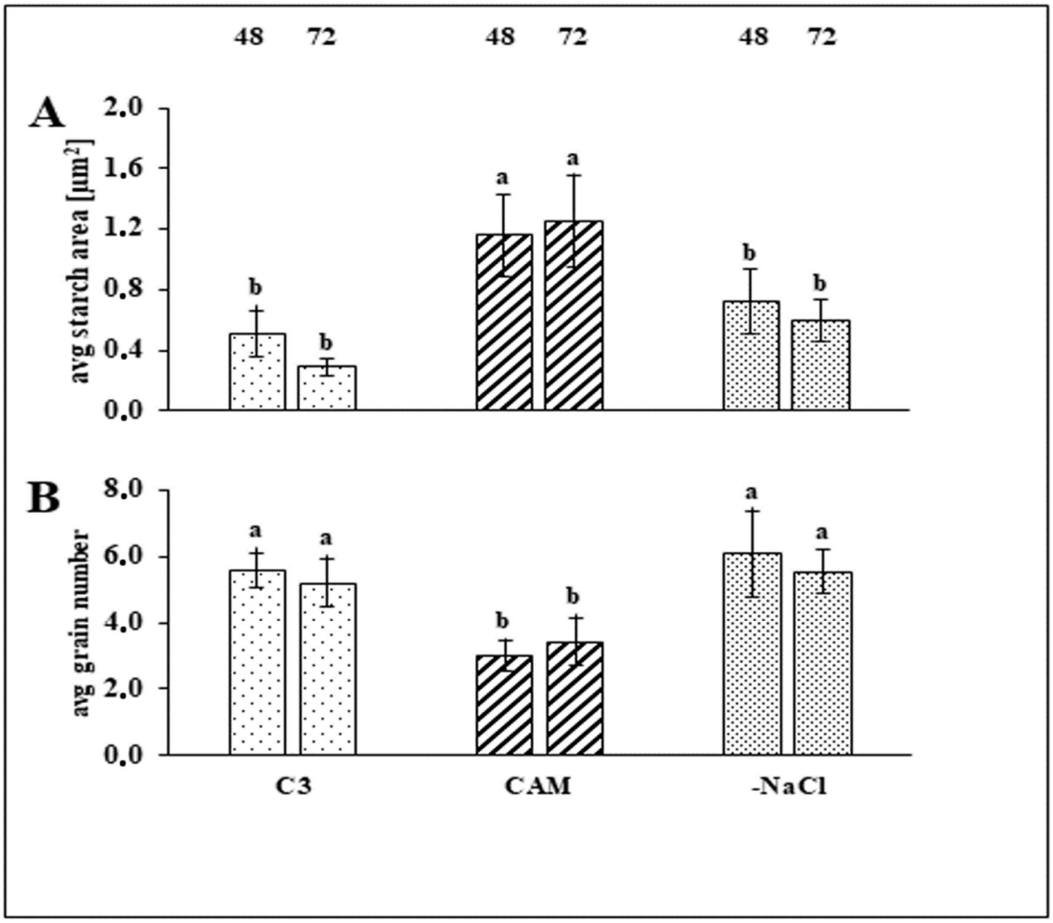

Figure S5. Second replication of the experiment. The average area (**A**) and number of starch grains (**B**) assessed with computer analysis of chloroplasts micrographs of unstressed control (C_3), NaCl-treated (CAM) and salt-stress withdrawn (-NaCl) *Mesembryanthemum crystallinum* L. plants 48 and 72 hours past osmotic stress. Bars represent mean values (±SD) for *n*=23. Different letters indicate statistically significant differences according to Tukey's HSD test at $p \leq 0.05$.

Table S1. Sequences of primers used during qPCR analysis.

Name	Accession number	Sequence (5′–3′)
PSI-A core protein of PS I	NC_029049.1 :40297-42549	F: TGAATGTGGTGGACCAAAAA R: CGTCCCCAAGATATGTTTTCA
PSI-B core subunit of PS I	AT5G01490 : 38067-40271	F: ATACTGTGCCCAATCCCAAA R: GCTCAAAATCCCGATTCAAG
D_1 protein of PS II	NC_029049.1: :518-1579	F: GGAAGATCAATCGACCGAAA R: CCTTATGCACCCATTTCACA
D_2 protein of PS II	NC_029049.1 :33519-34580	F: CTGTTCAATTGCGCCCTTAT R: CGTGAATAGCGCATAGCAAA
photosystem II core antenna protein CP43	NC_029049.1 :34528-35949	F: CTTTATTTTGGGGGCGTGTA R: GAGCCCATGCAAAAGGTTTA
UBQ – UBIQUITINE	AF053563	F: CGCACCTTGGCTGACTACA R: AACAACCAGACCATGCAACA
